# BNT162b2 Vaccination Elicits Strong Serological Immune Responses Against SARS-CoV-2 Including Variants of Concern in Elderly Convalescents

**DOI:** 10.3389/fimmu.2021.743422

**Published:** 2021-09-29

**Authors:** Bernd Jahrsdörfer, Dorit Fabricius, Judith Scholz, Carolin Ludwig, Aline Grempels, Ramin Lotfi, Sixten Körper, Guido Adler, Hubert Schrezenmeier

**Affiliations:** ^1^ Department of Transfusion Medicine, Ulm University, Ulm, Germany; ^2^ Institute for Clinical Transfusion Medicine and Immunogenetics, German Red Cross Blood Transfusion Service Baden-Württemberg – Hessen and University Hospital Ulm, Ulm, Germany; ^3^ Department of Pediatrics and Adolescent Medicine, Ulm University Medical Center, Ulm, Germany; ^4^ Medical Faculty, Ulm University, Ulm, Germany

**Keywords:** COVID-19, mRNA vaccine, Comirnaty, neutralization test, B.1.1.7, B.1.351, P.1, BNT162b2

## Abstract

Elderly residents of long-term care facilities (LTCFs) have long been underrepresented in studies on vaccine efficacy, particularly in light of currently emerging variants of concern (VOCs). In this prospective observational cohort study, we analyzed serological immune responses in 190 individuals before, 3 weeks after 1^st^ and 3 weeks after 2^nd^ vaccination with BNT162b2. Unvaccinated COVID-19-convalescent subjects served as reference. End points comprised serum anti-spike IgG and IgA titers as well as neutralization capacities against unmutated and mutated SARS-CoV-2 receptor binding domains including B.1.1.7, B.1.351 and P.1. We found that antibody titers and neutralization capacities up to 3 weeks after 2^nd^ vaccination with BNT162b2 were significantly higher in COVID-19-convalescent as compared to COVID-19-naive vaccinees. Moreover, pre-vaccination anti-NCP IgG titers, but not age or gender, had a high impact on the strength and kinetics of post-vaccination neutralization capacity development. Most importantly, BNT162b2-induced neutralization capacity was cross-reactive with VOCs. In contrast to unvaccinated convalescents, vaccinated convalescent individuals of all ages acquired strong neutralizing capacities against current VOCs. The present study suggests that COVID-19-convalescent individuals with a broad age range between 18 and 98 years benefit from BNT162b2 vaccination by developing strong and broad neutralizing immune responses against SARS-CoV-2 including current VOCs.

## Introduction

A number of studies provide evidence for the development of a rapid and efficient anti-SARS-CoV-2 immune response after single dose vaccination against SARS-COV-2 in COVID-19-convalescent individuals (1–6). Most of these studies enrolled health care professionals (HCPs) with a median age between 35 and 45 years. Among the most vulnerable populations in the context of COVID-19 are residents of long-term care facilities (LTCFs), mainly aged above 70 years, who had the highest incidence of infection and were among the first to be vaccinated. Although the impact of comorbidities, e.g. obesity (7, 8) or kidney diseases (9, 10) on serological immune responses after COVID-19 vaccination has been demonstrated in a series of cohort studies, the group of older individuals with multiple co-morbidities and frailty have long been underrepresented in most studies on the efficacy and safety of vaccines (11, 12). A population-level observational study from Denmark reported data on protection against SARS-CoV-2 repeat infections from a large cohort of more than 500.000 (13). Protective immunity was estimated to be 80-83% in individuals younger than 65 years. In contrast, in individuals aged 65 years and older estimated immunity was only about 47%. The authors concluded that previously infected elderly individuals need to be vaccinated since their natural immunity against reinfection was low. After launch of the Danish immunization program against SARS-CoV-2, a second observational cohort study with more than 39.000 LTCF residents revealed that indeed a two-dose regimen with the mRNA vaccine BNT162b2 provided a high protective effect also in elderly individuals (14).

Nevertheless, little is still known about immune responses in elderly individuals who recovered from COVID-19 as compared to COVID-19-negative individuals or individuals with a longer interval to their previous infection. This comparison is particularly important in the context of currently emerging SARS-CoV-2 variants of concern (VOCs), particularly B.1.351 and P.1 (15–17). Primary aim of the present study was to assess the dynamics of humoral immune responses before and after first and second vaccination with BNT162b2 in individuals of different age and post-COVID-19 immune status. A particular focus was set on neutralization capacities against VOCs as compared to the unmutated virus. Of note, our study was not powered to allow conclusions on the impact of demographic and clinical data. In total, we analyzed 190 residents and HCPs from five LCTFs with COVID-19 outbreaks within the last year.

## Materials and Methods

### Study Cohorts

The AnCORIm (“**An**tikörperantwort **CO**VID-19-**R**ekonvaleszenter nach **Im**pfung”) study is a prospective observational cohort study. It was conducted from March to May 2021 in five long-term care facilities (LCTFs), in which up to 90% of residents and up to 70% of health care professionals (HCPs) had prior SARS-COV-2 infections between January 2020 and February 2021. Participants were enrolled when they had a negative pharyngeal swab nucleic acid amplification technique (NAT) test result, were out of quarantine and had no clinical signs of COVID-19 disease. After oral explanation of risks and benefits of vaccination and demonstration of the study protocol, written informed consent was obtained from all participants or the persons appointed to make medical decisions on their behalf. The use of blood from study subjects before and after vaccination against SARS-CoV-2 as well as from COVID-19-convalescent subjects was approved by the Ethical Committee at Ulm University. A total of 203 individuals from five LCTFs, who received vaccination with BNT162b2, were recruited. Only vaccinees without history of diseases or medication affecting systemic immunity were included. 13 individuals dropped out, so that for 190 individuals (93.6%) relevant data were available for analysis. For analyses regarding neutralization capacity against VOCs we included an additional “historic” (2020) reference cohort of unvaccinated COVID-19-convalescent donors (18, 19). For age and gender characteristics of all vaccinated and unvaccinated individuals included see **Table 1**.

**Table 1:** Age, gender and anti-SARS-CoV-2-NCP IgG titer characteristics of study subjects.

(Sub-) cohorts	Number (%)	Age [median (range)]	Female [number (%)]	Male [number (%)]	Pre-vaccination anti-NCP IgG (mean OD ratio ± SD, cutoff = 1.1)
Total vaccinees	190 (100)	63 (18 – 98)	152 (80)	38 (20)	2.55 ± 0.16
COVID-19-convalescent (CC)	163 (86)	64 (20 – 98)	130 (80)	33 (20)	2.87 ± 0.17
COVID-19-naive (CN)	27 (14)	59 (18 – 94)	22 (81)	5 (19)	0.46 ± 0.18
NCP-pos.	112 (59)	53 (18 – 96)	88 (79)	24 (21)	3.88 ± 0.16
NCP-neg.	78 (41)	76 (20 – 98)	64 (82)	14 (18)	0.41 ± 0.04
Age < 64	97 (51)	49 (18 – 63)	81 (84)	16 (16)	1.73 ± 0.18
Age ≥ 64	93 (49)	85 (64 – 98)	71 (76)	22 (24)	3.43 ± 0.25
Female	152 (80)	60 (18 – 98)	152 (100)	0 (0)	2.42 ± 0.18
Male	38 (20)	71 (19 – 94)	0 (0)	38 (100)	2.91 ± 0.36
NCP-neg., age < 64	51 (27)	47 (18 – 63)	44 (86)	7 (14)	0.41 ± 0.05
NCP-neg., age ≥ 64	22 (12)	83 (64 – 96)	16 (73)	6 (27)	0.41 ± 0.07
NCP-pos., age < 64	46 (24)	51 (20 – 63)	38 (83)	8 (17)	3.27 ± 0.22
NCP-pos., age ≥ 64	71 (37)	87 (64 – 98)	54 (76)	17 (24)	4.42 ± 0.23
Unvaccinated COVID-19-convalescents	20	44 (24 - 62)	10 (50%)	10 (50%)	3.33 ± 0.41

A total of 203 individuals undergoing vaccination against SARS-CoV-2 using the mRNA vaccine BNT162b2 were recruited in long-term care facilities including 48% residents and 52% healthcare professionals. Complete data were available from 190 (93.6%) of these individuals. A reference cohort consisting of 20 unvaccinated COVID-19-convalescent individuals served as reference for testing of neutralization capacity against variants of concern. NCP, nucleocapsid; OD, optical density.

### Serum Isolation and Cryopreservation

For serologic and neutralization testing, up to 20ml blood from each donor was collected in serum collection tubes with clot activator (Vacuette, Greiner Bio-One GmbH, Frickenhausen, Germany) prior to first vaccination, 21 days later prior to second vaccination and 21 days after second vaccination of the BNT162b2 mRNA vaccine. Serum collection tubes were centrifuged at 1500g and 20°C for 15min, aliquoted into 250µl-1000µl aliquots in 2ml cryopreservation tubes (Greiner Bio-One GmbH, Frickenhausen, Germany) and cryopreserved at -20°C until further use. For long-term storage cryopreservation tubes were transferred into -80°C.

### Enzyme-Linked Immunosorbent Assays

The Euroimmun anti-SARS-CoV-2 ELISA assays (EUROIMMUN, Lübeck, Germany) were used for the detection of IgG and IgA to the S1 domain of the SARS-COV-2 spike (S) protein, and IgG to the SARS-CoV-2 nucleocapsid (NCP) protein. Briefly, serum or plasma samples were diluted at 1:101 in sample buffer and pipetted onto strips of 8 single wells of a 96-well microtiter plate, precoated with recombinant SARS-CoV-2 spike or nucleocapsid proteins. A calibrator, a positive control and a negative control were carried out on each plate. After incubation for 60 minutes at 37°C, wells were washed 3 times and the peroxidase-labeled anti-IgG or anti-IgA antibody solution was added, followed by a second incubation step for 30 min. After three additional washing steps, substrate solution was added and the samples incubated for 15 - 30 minutes in the dark. After adding the stop solution, optical density (OD) values were measured on a POLARstar Omega plate reader (BMG Labtech, Ortenberg, Germany) at 450 nm and at 620 nm. Finally, OD ratios were calculated based on the sample and calibrator OD values. For all analytes, a ratio < 0.8 was considered to be non-reactive or negative. An OD-ratio of ≥ 1.1 was considered to be positive for all three analytes. Samples with OD ratios >10 were prediluted in sample buffer at 1:10 - 1:50 and analyzed again. Results were extrapolated accordingly.

### Surrogate SARS-CoV-2 Neutralization Test (GenScript)

The principle of this blocking ELISA mimics the cellular docking process of the virus, thereby indirectly detecting anti-SARS-CoV-2 antibodies, which suppress the interaction between receptor binding domain (RBD) fragments of the viral spike (S) protein and the angiotensin-converting enzyme 2 (ACE2) protein bound to the surface of a microtiter plate (**Supplementary Figure 1**). After pre-incubation of samples and controls, which allows antibodies in the serum to bind to a horseradish peroxidase (HRP)-conjugated RBD fragment (HRP-RBD), the mixture was added to a capture plate coated with human ACE2 protein. Any unbound HRP-RBD or HRP-RBD bound to non-neutralizing antibodies was captured on the plate. Then, three washing steps were performed to remove complexes of neutralizing antibodies and HRP-RBD that did not bind to the plate. Subsequently, TMB was added as a substrate, allowing HRP to catalyze a color reaction. Color change from blue to yellow after addition of the stop reagent was read on a microtiter plate reader at 450nm (OD450). The absorbance of the sample is inversely correlated with the amount of SARS-CoV-2 neutralizing antibodies. Positive and negative controls serve as internal assay quality controls. The test is considered valid only if the OD450 for each control falls within the respective range (OD450_negative control_ > 1.0, OD450_positive control_ < 0.3). For final interpretation, inhibition rates were calculated as follows: Inhibition score (%) = (1 - (OD value_sample_/OD value_negative control_) x 100%). Scores < 30% were considered to be negative, scores ≥ 30% were considered to be positive. To investigate the capacity of neutralizing antibodies to cover certain variants of concern (VOCs), different modifications of HRP-RBD were used: SARS-CoV-2-spike RBD N501Y (B.1.1.7), SARS-CoV-2-spike RBD E484K, K417N, N501Y (B.1.351) and SARS-CoV-2-spike RBD E484K, K417T, N501Y (P.1). The assay protocol was followed as described above, however, to allow better discriminiation between wildtype and variant RBDs, serum samples were pre-diluted at 1:20 and were tested in parallel using wildtype HRP-RBD and the three HRP-RBD variants. The obtained results were corrected based on the results obtained using the wildtype HRP-RBD without dilution.

### Statistical Analysis

Statistical analysis was performed using Microsoft Excel for Mac, version 16.16.8 and GraphPad Prism, version 9.0.0. Summarized data in line graphs are expressed as box plots with central horizontal lines showing medians, box edges representing interquartile ranges and whiskers representing the minima and maxima. Dunnetts’s multiple comparisons test was performed for comparison of neutralization capacities against VOCs in vaccinated subcohorts versus the unvaccinated convalescent reference cohort. Tukey´s test for multiple comparisons was used for comparison of more than two independent data sets and more than two variables. Mann-Whitney U test was used for comparison of two data sets. Spearman correlation was used to assess associations between neutralization capacity and anti-spike titers.

## Results

### Characteristics of Study Cohort and Post-Vaccination Symptoms

For the present study, a total of 203 individuals including 48% residents and 52% healthcare professionals (HCPs) from five long-term care facilities were recruited and serological immune responses monitored in the course of vaccination with the mRNA vaccine BNT162b2. Complete data were available from 190 individuals (94%) and were analyzed after grouping into various subcohorts. Details on subcohorts, age and gender characteristics are summarized in **Table 1**. 163 individuals (85.8%) had a documented history of COVID-19 with positive pharyngeal swab NAT testing (subcohort CC), whereas 27 individuals (14.2%) never had a positive test result and did not report any COVID-19-specific symptoms since the beginning of the pandemic (subcohort CN). Overall, vaccinations were well tolerated in all participants with no severe side effects observed within 60min after vaccination or during the following days. After 1^st^ vaccination, 11% of residents reported injection site symptoms, fatigue or fever, while in the group of HCPs 33% reported injection site symptoms, muscle symptoms, fatigue, headache, fever, chills and lymph node swelling. After 2^nd^ vaccination, only 4% of residents and 20% of HCPs reported symptoms.

### Antibody Titers and Neutralization Capacities Before and After Vaccination With BNT162b2 Are Significantly Higher in COVID-19-Convalescent Than in -Naive Vaccinees

**Figure 1:**
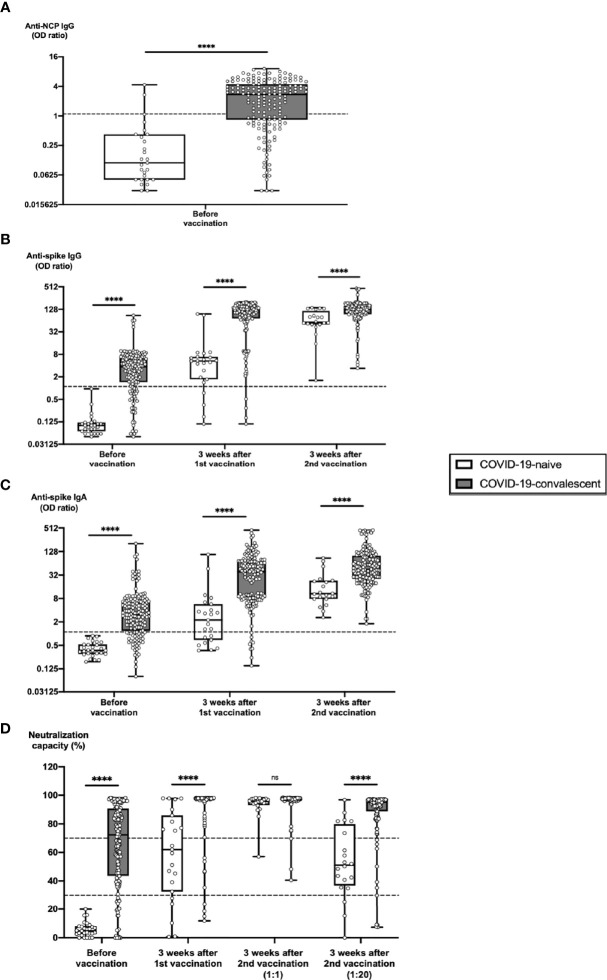
Humoral SARS-CoV-2-specific immune parameters after vaccination of COVID-19-naive versus -convalescent individuals. Serum samples from 163 subjects with confirmed COVID-19 history (COVID-19-convalescent, CC) and from 27 subjects without such history (COVID-19-naive, CN) were collected before vaccination, 3 weeks after first vaccination and 3 weeks after second vaccination with BNT162b2. Samples were analyzed for anti-NCP IgG titers, anti-spike IgG and IgA titers as well as for ACE2-RBD neutralization capacities. Box blots show **(A)** anti-NCP IgG titers, **(B)** anti-spike IgG titers, **(C)** anti-spike IgA titers and **(D)** median neutralization capacities at different time points as indicated. Dotted lines indicate cutoff values. To further differentiate high neutralization capacities 3 weeks after second vaccination, an additional analysis using an extra serum pre-dilution of 1:20 was performed (blot pair on the right side in panel D). Box central horizontal lines indicate medians, box borders represent IQR, whiskers indicate minima and maxima. Significance level was ****p < 0.00005. ACE2, angiotensin-converting enzyme 2; IQR, interquartile ranges; NCP, nucleocapsid; ns, not significant; OD, optical density; RBD, receptor-binding domain.

As expected, anti-NCP IgG titers were significantly higher in the COVID-19-convalescent subcohort (CC) compared with the COVID-19-negative subcohort (CN) (**Figure 1A**). Nevertheless, 3 subjects (11%) in subcohort CN exhibited positive anti-NCP IgG levels, suggesting the presence of asymptomatic infections in this subcohort. Vice versa, 52 individuals (31.9%) in subcohort CC exhibited negative anti-NCP IgG titers.

Likewise, mean antibody titers against the spike protein were significantly higher in subcohort CC than in subcohort CN before vaccination (**Figures 1B, C**). This was true for both anti-spike IgG titers as well as anti-spike IgA titers, which represent an additional important first line of virus defense and which correlate with viral immunity on mucosal surfaces. After 1^st^ vaccination, there was a strong enhancement in anti-spike antibody titers in both subcohorts, with comparable increases in subcohort CN (53.7-fold for anti-spike IgG and 16.1-fold for anti-spike IgA) and subcohort CC (59.9-fold for anti-spike IgG and 15.7-fold for anti-spike IgA). In contrast, after 2^nd^ vaccination additional increases in anti-spike antibody titers were significantly higher in subcohort CN (19.1-fold for anti-spike IgG and 6.9-fold for anti-spike IgA) than in subcohort CC (4.5-fold for anti-spike IgG and 4.4-fold for anti-spike IgA). Nevertheless, mean antibody titers were still significantly higher 3 weeks after 2^nd^ vaccination in subcohort CC as compared to subcohort CN (1.8-fold for anti-spike IgG and 4.0-fold for anti-spike IgA). Moreover, a strong overall correlation could be observed between anti-spike IgG and IgA titers (**Supplementary Figure 2A**).

Similar observations were made with RBD-ACE2 neutralization capacity in both cohorts (**Figure 1D**). Before vaccination, 0% of individuals in subcohort CN versus 86.1% of individuals in subcohort CC had detectable neutralization capacity (cutoff 30%). 3 weeks after 1^st^ vaccination, >78% of vaccinees in subcohort CN had detectable and >43% of vaccinees had strong neutralization capacity (cutoff 70%), compared with >97% of vaccinees in subcohort CC with detectable and > 93% with strong neutralization capacity. At 3 weeks after 2^nd^ vaccination, 100% of vaccinees in both subcohorts had neutralization capacity detectable with no significant difference between both subcohorts. Nevertheless, additional 1:20 pre-dilution of serum samples revealed that individuals in subcohort CC still had a highly significant advantage regarding neutralization capacity over subcohort CN. As in unvaccinated convalescent individuals (18, 19), we could confirm that in BNT162b2-vaccinated individuals anti-spike IgG titers strongly correlated with SARS-CoV-2 neutralization capacity, although 3 weeks after 1^st^ vaccination in a small number of individuals with high neutralization capacity (2%) anti-spike IgG and IgA titers still ranged below the cutoff value (**Supplementary Figure 2B**).

**Figure 2:**
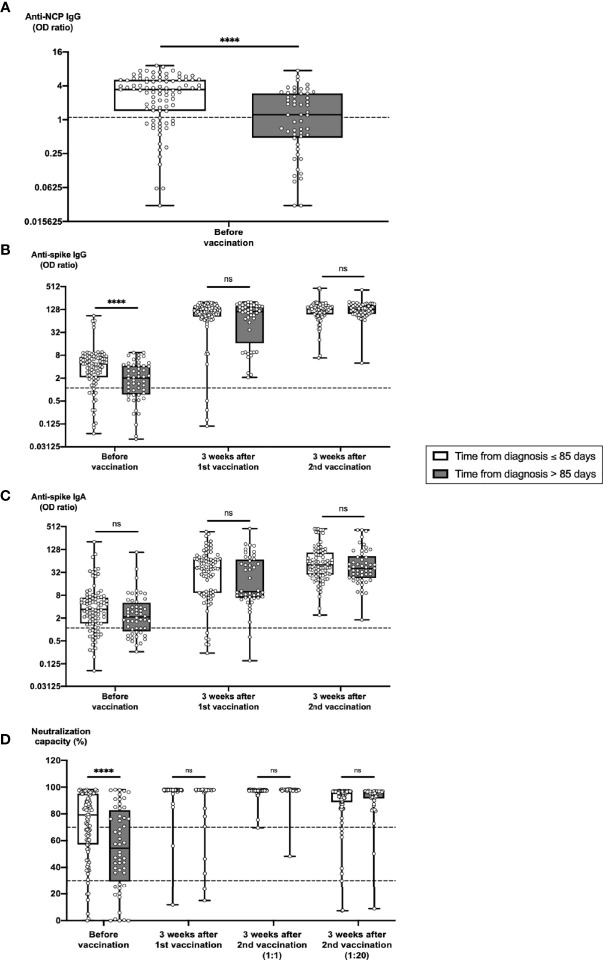
Impact of time from COVID-19 diagnosis on the development of humoral immune parameters after vaccination. Serum samples from 91 subjects with a time from diagnosis ≤ 85 days and from 52 subjects with a time from diagnosis > 85 days were collected before vaccination, 3 weeks after first vaccination and 3 weeks after second vaccination with BNT162b2. Samples were analyzed for anti-spike IgG and IgA titers, anti-NCP IgG titers as well as for ACE2-RBD neutralization capacities. Box blots show **(A)** anti-NCP IgG titers, **(B)** anti-spike IgG titers, **(C)** anti-spike IgA titers and **(D)** median neutralization capacities at different time points as indicated. Dotted lines indicate cutoff values. To further differentiate high neutralization capacities 3 weeks after second vaccination, an additional analysis using an extra serum pre-dilution of 1:20 was performed (blot pair on the right side in panel D). Box central horizontal lines indicate medians, box borders represent IQR, whiskers indicate minima and maxima. Significance level was ****p < 0.00005. ACE2, angiotensin-converting enzyme 2; IQR, interquartile ranges; NCP, nucleocapsid; ns, not significant; OD, optical density; RBD, receptor-binding domain.

To elucidate whether the time from diagnosis might impact on the pace and intensity of serological responses to BNT162b2 vaccination, we split subcohort CC into two groups, one with a time from diagnosis ≤ 85 days (average 70, range 11 – 85 days) and the other with a time from diagnosis > 85 days (average 163, range 91 – 444 days). Before vaccination, individuals with shorter intervals from diagnosis exhibited significantly stronger levels of anti-NCP IgG (**Figure 2A**) and anti-spike IgG (**Figure 2B**) than individuals with longer intervals from diagnosis, whereas anti-spike IgA titers did not significantly differ between both groups (**Figure 2C**). Differences in neutralization capacities were comparable to anti-spike IgG titers before vaccination with 59% of individuals with shorter intervals from diagnosis versus 37% of individuals with longer intervals from diagnosis exhibiting strong neutralization capacity (cutoff 70%) (**Figure 2D**). Serological differences between both groups disappeared beyond 3 weeks after 1^st^ vaccination (**Figures 2B–D**).

### Strong Neutralizing Immune Responses to BNT162b2 Develop Faster and Are Significantly Stronger in Individuals With Positive Pre-Vaccination Anti-NCP IgG Titers, but Do Not Depend on Age or Gender

**Figure 3:**
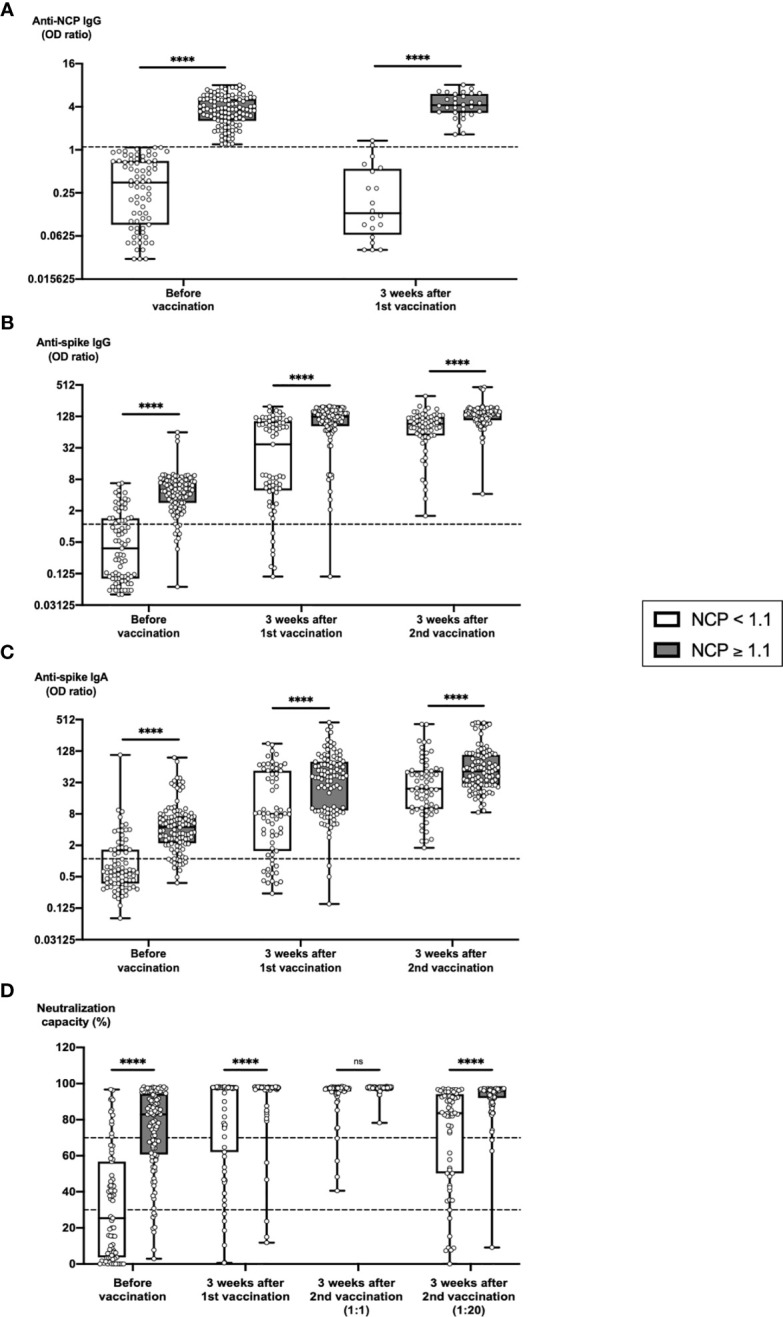
Predictive value of pre-vaccination anti-NCP IgG titers on the development of humoral immune parameters after vaccination. Serum samples from 75 subjects with anti-NCP IgG OD ratios < 1.1 and from 112 subjects with anti-NCP IgG OD ratios ≥ 1.1 were collected before vaccination, 3 weeks after first vaccination and 3 weeks after second vaccination with BNT162b2. Samples were analyzed for anti-spike IgG and IgA titers, anti-NCP IgG titers as well as for ACE2-RBD neutralization capacities. Box blots show **(A)** anti-NCP IgG titers, **(B)** anti-spike IgG titers, **(C)** anti-spike IgA titers and **(D)** median neutralization capacities at different time points as indicated. Dotted lines indicate cutoff values. To further differentiate high neutralization capacities 3 weeks after second vaccination, an additional analysis using an extra serum pre-dilution of 1:20 was performed (blot pair on the right side in panel D). Box central horizontal lines indicate medians, box borders represent IQR, whiskers indicate minima and maxima. Significance level was ****p < 0.00005. ACE2, angiotensin-converting enzyme 2; IQR, interquartile ranges; NCP, nucleocapsid; ns, not significant; OD, optical density; RBD, receptor-binding domain.

To exclude a potential bias of our results by relying on anamnestic information and SARS-CoV-2 NAT results only, we used an additional stratification based on anti-NCP responses as representative marker of the post-COVID-19 immune status. We evaluated the impact of anti-NCP IgG titers before vaccination on the dynamics of the serological immune response to BNT162b2 vaccination by splitting the study cohort into two subcohorts based on pre-vaccination anti-NCP IgG levels (**Figure 3A**, left panel side). Up to 3 weeks after 1^st^ vaccination anti-NCP IgG levels remained stable in both subcohorts (**Figure 3A**, right panel side). In contrast, anti-spike antibody IgG and IgA titer were strongly boosted after 1st vaccination (**Figures 3B, C**). Individuals in the NCP-negative subcohort exhibited an 89.8-fold increase for anti-spike IgG and a 19.7-fold increase for IgA, individuals in the NCP-positive subcohort a 32.0-fold increase for anti-spike IgG and a 14.1-fold increase for anti-spike IgA. After 2^nd^ vaccination, additional increases in anti-spike antibody titers were significantly higher in the NCP-negative subcohort (12.7-fold for anti-spike IgG and 5.1-fold for anti-spike IgA) compared with the NCP-positive subcohort (2.4-fold for anti-spike IgG and 4.6-fold for anti-spike IgA). Eventually, 3 weeks after 2^nd^ vaccination, mean antibody titers reached significantly higher levels in the NCP-positive compared with the NCP-negative subcohorts (1.6-fold for anti-spike IgG and 1.7-fold for anti-spike IgA).

Similarly, significantly higher numbers of NCP-positive versus NCP-negative vaccinees (95.2% versus 73.1%) reached strong neutralization capacity 3 weeks after 1^st^ vaccination (**Figure 3D**). 3 weeks after second vaccination >93% of vaccinees in the NCP-negative and 100% of vaccines in the NCP-positive subcohort had reached strong neutralization capacity. Although this difference did not reach statistical significance, an additional 1:20 pre-dilution of serum samples again revealed that neutralization capacity in individuals from the NCP-positive subcohort were indeed still significantly stronger than in individuals from the NCP-negative subcohort.

**Figure 4:**
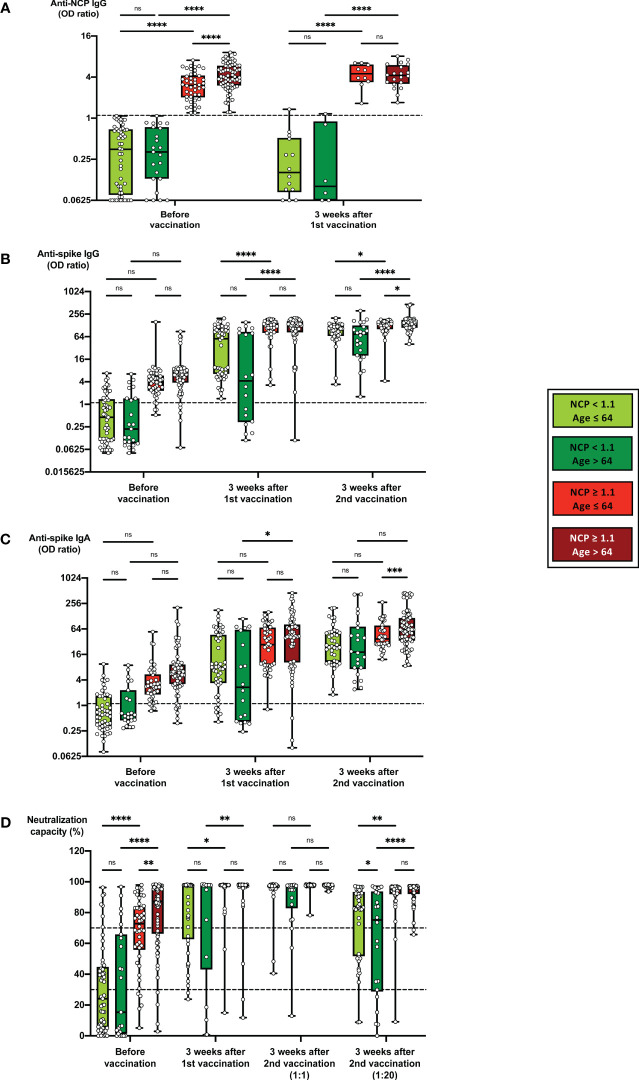
Impact of age on the development of humoral immune parameters after vaccination. Serum samples from 51 subjects with anti-NCP IgG OD ratios < 1.1 and an age < 64, from 22 subjects with anti-NCP IgG OD ratios < 1.1 and an age ≥ 64, from 46 subjects with anti-NCP IgG OD ratios ≥ 1.1 and an age < 64 and from 71 subjects with anti-NCP IgG OD ratios ≥ 1.1 and an age ≥ 64 were collected before vaccination, 3 weeks after first vaccination and 3 weeks after second vaccination with BNT162b2. Samples were analyzed for anti-spike IgG and IgA titers, anti-NCP IgG titers as well as for ACE2-RBD neutralization capacities. Box blots show **(A)** anti-NCP IgG titers, **(B)** anti-spike IgG titers, **(C)** anti-spike IgA titers and **(D)** median neutralization capacities at different time points as indicated. Dotted lines indicate cutoff values. To further differentiate high neutralization capacities 3 weeks after second vaccination, an additional analysis using an extra serum pre-dilution of 1:20 was performed (blot pair on the right side in panel D). Box central horizontal lines indicate medians, box borders represent IQR, whiskers indicate minima and maxima. Significance levels were *p < 0.05, **p < 0.005, ***p < 0.0005 and ****p < 0.00005. ACE2, angiotensin-converting enzyme 2; IQR, interquartile ranges; NCP, nucleocapsid; ns, not significant; OD, optical density; RBD, receptor-binding domain.

An additional subgroup analysis was performed to identify the potential impact of age on the serological response to BNT162b2 vaccination (**Figure 4**). Before vaccination, significantly higher anti-NCP IgG titers (**Figure 4A**) and stronger neutralization capacities (**Figure 4D**) were detected in older as compared to younger NCP-positive individuals, which is in line with the observation of a higher prevalence of COVID-19 convalescence in older individuals (**Table 1**). Nevertheless, 3 weeks after 1^st^ vaccination, any differences for serological parameters between younger and older vaccinees had disappeared (**Figure 4**). 3 weeks after 2^nd^ vaccination again, anti-spike IgG and IgA titers, but not neutralization capacities ranged significantly higher in older than in younger NCP-positive individuals (**Figures 4B–D**). This was in line with the finding that the overall increase of anti-spike IgG and IgA titers in the course of BNT162b2 vaccination was significantly higher in older as compared to younger NCP-positive vaccinees (**Supplementary Figures 3 and 4**).

Finally, a gender-dependent analysis of serological results before and after vaccination revealed no significant gender-specific differences in anti-NCP IgG titers, anti-spike IgG and IgA titers or neutralization capacities (**Supplementary Figure 5**).

### BNT162b2-Induced Neutralizing Immune Responses Against the Currently Most Widespread Variants of Concern Are Significantly Stronger in Individuals With Positive Than With Negative Anti-NCP IgG Pre-Vaccination Titers

**Figure 5:**
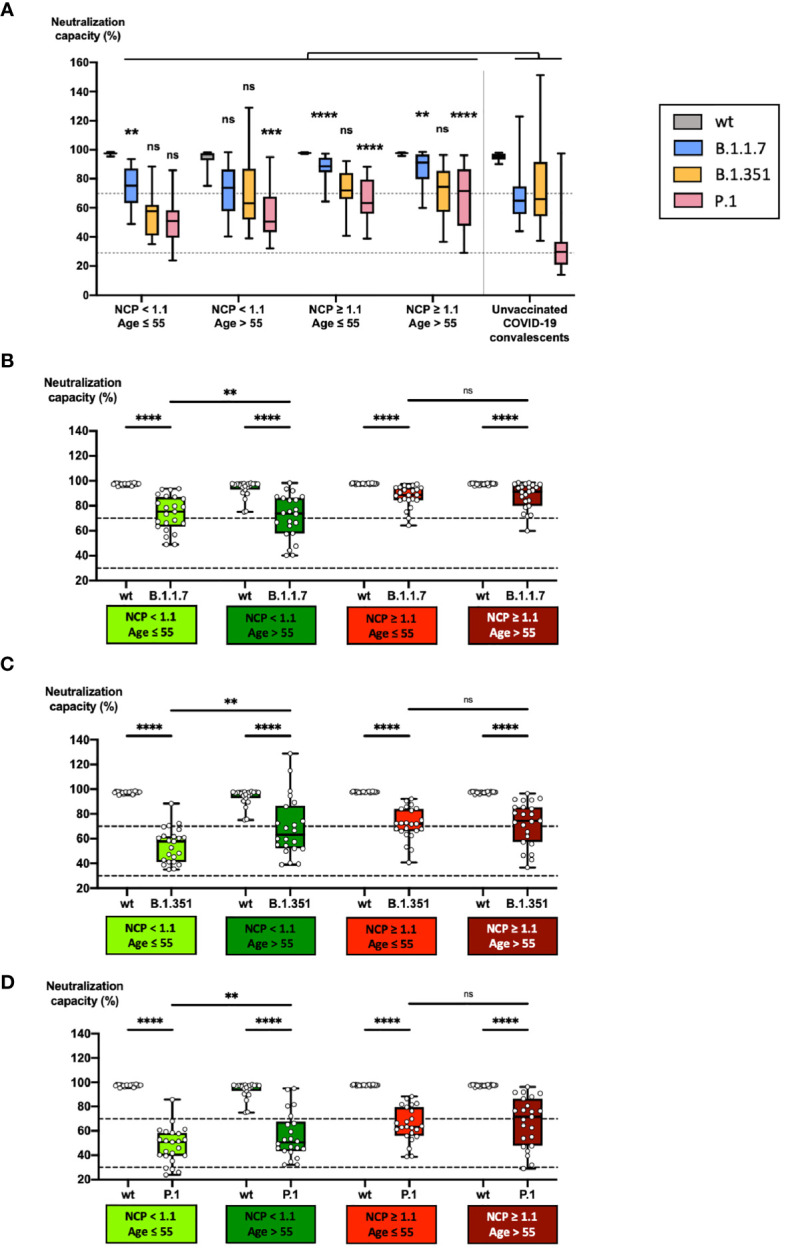
Impact of age and COVID-19 convalescence on neutralizing capacity against variants of concern after vaccination. Serum samples from 23 subjects with anti-NCP IgG OD ratios < 1.1 and an age > 55, from 23 subjects with anti-NCP IgG OD ratios < 1.1 and an age ≤ 55, from 23 COVID-19-convalescent subjects with anti-NCP IgG OD ratios ≥ 1.1 and an age > 55 and from 23 OVID-19-convalescent subjects with anti-NCP IgG OD ratios ≥ 1.1 and an age ≤ 55 were collected 3 weeks after second vaccination with BNT162b2. Serum samples from 20 unvaccinated COVID-19-convalescent individuals, collected at a median time of 14 weeks after diagnosis served as control cohort. Samples were pre-diluted at 1:20 and analyzed for neutralization capacities against wildtype RBD and three RBD variants of concern. Box plots in panel **(A)** compare neutralization capacities in the four vaccinated cohorts with those in the unvaccinated COVID-19-convalescent cohort. Box blots in panels **(B–D)** show neutralization capacities against wildtype RBD and **(B)** the B.1.1.7 RBD variant, **(C)** the B.1.351 RBD variant and **(D)** the P.1 RBD variant. Dotted lines indicate cutoff values. Box central horizontal lines indicate medians, box borders represent IQR, whiskers indicate minima and maxima. Significance levels were **p < 0.005, ***p < 0.0005 and ****p < 0.00005. IQR, interquartile ranges; NCP, nucleocapsid; ns, not significant; OD, optical density; RBD, receptor-binding domain; wt:, wildtype (unmutated).

Of particular interest was the question, which subcohorts may benefit most from vaccination with regard to the currently emerging SARS-CoV-2 variants of concern (VOCs), older versus younger individuals, and COVID-19-convalescent subjects with positive anti-NCP IgG titers versus subjects with negative anti-NCP IgG titers. We therefore compiled 4 subcohorts based on age and pre-vaccination anti-NCP IgG titer, and tested them for neutralization capacity against the 3 VOCs B.1.1.7, B.1.351 and P.1 (**Figure 5**). As a general observation, a gradual loss of neutralizing potency when comparing the unmutated SARS-CoV-2 with the three VOCs tested was evident, both in the vaccinated four subcohorts as well as in an unvaccinated COVID-19-convalescent reference cohort (**Figure 5A**). In line with this observation, neutralization capacities against all VOCs were significantly lower compared with neutralization capacities against the original virus, regardless of age or anti-NCP IgG titers before vaccination (**Figures 5B–D**).

Compared with unvaccinated COVID-19-convalescent individuals, vaccinated convalescent individuals of both age groups showed significantly stronger average neutralizing capacities against B.1.1.7 (88%) and P.1 (65% - 67%) (**Figure 5A**). In contrast, younger individuals with negative pre-vaccination anti-NCP IgG titers had a significantly stronger average neutralization capacity against B.1.1.7 (71%) (**Figure 5B**) and older individuals against P.1 (56%) (**Figure 5D**) compared with unvaccinated convalescent individuals (63% against B.1.17, 34% against P.1). Of note, average neutralization capacities against the variant B.1.351 was not significantly stronger in any of the vaccinated subcohorts compared with unvaccinated convalescent individuals (70% against B.1.351) and ranged between 55% and 72% (**Figure 5A**). After vaccination, neutralization capacities against all variants tested were significantly stronger in convalescent individuals (anti-NCP IgG ≥ 1.1) as compared to individuals with anti-NCP IgG titers < 1.1, regardless of age (**Figures 5B–D**). Within the subcohort of vaccinated convalescent individuals (anti-NCP IgG ≥ 1.1), anti-VOC neutralization capacities were not significantly different between younger (88% against B.1.1.7, 72% against B.1.351, 65% against P.1) and older (88% against B.1.1.7, 72% against B.1.351, 67% against P.1) individuals. In contrast, vaccinated older individuals with pre-vaccination anti-NCP IgG titers < 1.1 developed significantly stronger neutralization capacities against B.1.351 (69%) and P.1. (56%) compared with younger individuals with equal anti-NCP status (55% against B.1.351, 48% against P.1) (**Figures 5C, D**).

## Discussion

The most important pillar in the fight against the SARS-CoV-2 pandemic is vaccination with the main intention to prevent severe infection courses and reinfections by variants of concern (VOCs). Meanwhile, we know that neutralizing antibody levels are highly predictive of immune protection from symptomatic SARS-CoV-2 infection, disease severity and survival (20, 21). Despite long-term memory of both neutralizing serological and cellular immunity to SARS-CoV-2 infection after COVID-19 (18, 22–24), large observational cohort studies have demonstrated that also in COVID-19-convalescent individuals, vaccination can efficiently prevent reinfections (13, 14). Furthermore, several studies have shown that in individuals with previous SARS-CoV-2 infections even a single dose vaccination elicits robust and efficient antibody responses (1–6) and may even boost cross-variant neutralizing antibodies as shown for the mutation B.1.351 (25). Nevertheless, a detailed analysis of neutralizing antibody responses in elderly convalescent individuals including residents of long-term care facilities (LTCFs) is lacking so far, particularly in the light of emerging VOCs (17).

The current study demonstrates that the above-mentioned potentiation of anti-SARS-CoV-2-specific serological responses in COVID-19-convalescent individuals compared with COVID-19-naïve individuals does not only occur in younger subjects, but also in elderly individuals up to an age of 98 years. Eighty-six percent of COVID-19-convalescent individuals versus 0% of COVID-19-negative individuals started out with detectable neutralization capacities > 30% before vaccination. Although the difference between both groups continuously disappeared until 3 weeks after 2^nd^ vaccination, anti-spike antibody titers were still 2-4 times higher and neutralization capacities against the unmutated virus was almost twice as strong in COVID-19-convalescent compared with COVID-19-negative individuals. Importantly, the interval between SARS-CoV-2 infection and vaccination did not affect the extent of the described responses, confirming that immune memory did not significantly decrease within the observed temporal range of up to one year between SARS-COV-2 infection and vaccination (26).

A more detailed subcohort analysis allowed an estimation of the impact of age and gender on the development of antibody titers and neutralization capacities after vaccination. To exclude a potential bias by relying on anamnestic information and SARS-CoV-2 NAT results only, we established four subcohorts based on age and pre-vaccination anti-NCP IgG levels. Comparison of these subcohorts revealed highly significant differences in anti-spike IgG and IgA titers as well as in neutralization capacities against unmutated SARS-CoV-2 between anti-NCP IgG-positive and -negative individuals. 3 weeks after 2^nd^ vaccination anti-spike IgG and IgA titers in the anti-NCP IgG-positive subcohorts ranged between 60% and 70% higher compared to the anti-NCP IgG-negative subcohort. No age-dependent differences in antibody titers were found in anti-NCP IgG-negative individuals, whereas in convalescent individuals anti-spike IgG and IgA titers were significantly higher in older (> 64 years) as compared to younger (≤ 64 years) subjects. In contrast, neutralization capacities were comparably strong in both age groups, with 100% of vaccinees reaching strong neutralization capacities until 3 weeks after 2^nd^ vaccination, although further titration of the sera beyond a 1:20 dilution may have revealed latent differences between the groups. Finally, gender-specific analysis demonstrated that no significant differences existed between females and males regarding the development of SARS-CoV-2-specific antibody or neutralization titers after vaccination.

As described for neutralization capacities against the unmutated virus (wildtype, wt), those against VOCs ranged significantly higher in individuals with positive pre-vaccination anti-NCP IgG titers (convalescent individuals) compared with individuals with negative anti-NCP IgG titers. The European Centre for Disease Prevention and Control lists 3 currently relevant VOCs on its website, which have started superseding the VOC B.1.1.7 (Alpha, primarily described in the United Kingdom and predominant in the beginning of 2021): B.1.351 (Beta, primarily described in South Africa), P.1 (Gamma, primarily described in Brazil) and B.1.617.2 (Delta, primarily described in India) (https://www.ecdc.europa.eu/en/covid-19/variants-concern). As for the original SARS-CoV-2 wildtype, no significant differences were found between younger and older convalescent individuals with regard to neutralization capacities against B.1.1.7 (88% vs. 88%), B.1.351 (72% vs. 72%) and P.1 (65% vs. 67%) after vaccination. In contrast, subjects with negative pre-vaccination anti-NCP titers showed significant age differences, with younger individuals exhibiting lower neutralization capacities against B.1.351 (55%) and P.1 (48%), compared with older individuals (69% against B.1.351, 56% against P.1). Recently, the FDA updated their recommendations regarding the definition of high titer COVID-19 convalescent plasma, in which neutralization capacities > 68% with the neutralization assay used in the current study were considered sufficiently strong to induce beneficial effects in COVID-19 patients (https://www.fda.gov/media/141477/download). Although these recommendations primarily refer to neutralization of unmutated SARS-CoV-2, a threshold of 68% may also represent a valid point of reference for strong neutralization of VOCs. Our data therefore suggests that in contrast to unvaccinated convalescent subjects, convalescent individuals acquire strong neutralization capacities against the currently most widespread VOCs after full vaccination, regardless of their age. Our data confirms very recent findings that two vaccinations with BNT162b2 provide broad protection against VOCs regardless of age (27). In addition, our study demonstrates that this protection is significantly weaker in COVID-19-negative as compared to COVID-19-convalescent older individuals.

As a general note, interpretation of age- and gender-specific differences or similarities must be exercised with some caution. On the one hand, behavior differences with regard to infection control and prevention measures may have impacted on the frequency of previous infections with SARS-CoV-2 depending on age and gender. For example, our data indicate substantial differences between pre-vaccination anti-NCP IgG titers in older compared to younger individuals and in males compared to females, suggesting a potential bias on vaccination responses between these groups (**Table 1**). On the other hand, there was a 20% to 80% increase in mortality rates in elderly LTCF residents since the beginning of the SARS-CoV-2 pandemic, potentially resulting in a selection of elderly individuals with a generally more efficient immune response against SARS-CoV-2. Nevertheless, we believe that altogether the impact of a potential bias arising from the described effects is not strong enough to challenge the basic conclusions drawn from the data presented. Overall, our results support the basic conclusions from several large cohort studies, which demonstrated that after two vaccinations with BNT162b2, comparable and robust serological immune responses are achieved in individuals with a wide age range from 18 to 85 years (27–29).

In summary, the present prospective observational cohort study allows conclusions on the development of neutralizing immune responses after full vaccination with the mRNA vaccine BNT162b2 in a large cohort of COVID-19-convalescent individuals with a broad age range between 18 and 98 years. We demonstrated that antibody titers and neutralization capacities before and up to 3 weeks after 2^nd^ vaccination with BNT162b2 were significantly higher in COVID-19-convalescent as compared to COVID-19-naive vaccinees and that pre-vaccination anti-NCP IgG titers had a high predictive value with regard to strength and rapidity of post-vaccination neutralization capacity development. On the other hand, age or gender did not impact on the generation of neutralizing immune responses after vaccination of convalescent individuals. Most importantly, BNT162b2-induced neutralizing immune responses were also directed against the currently most widespread variants of concern. In contrast to unvaccinated COVID-19-convalescent individuals, convalescent individuals of all ages reached strong neutralizing capacities against the variants B.1.1.7, B.1.351 and P.1 after vaccination.

## Data Availability Statement

The raw data supporting the conclusions of this article will be made available by the authors, without undue reservation.

## Ethics Statement

The studies involving human participants were reviewed and approved by Ethics Committe of Ulm University. The patients/participants provided their written informed consent to participate in this study.

## Author Contributions

BJ, GA and HS designed the study and performed literature search. GA recruited donors and acquired samples. JS, CL, and AG performed the analytics. BJ and HS conceptualized and supervised the analytics. JS, CL, AG, and BJ collected data. BJ and DF analyzed and interpreted data and prepared figures. BJ, RL, SK, and HS provided key research tools. DF and BJ wrote the manuscript. BJ, DF, and CL verified the underlying data. All authors critically reviewed the manuscript. All authors contributed to the article and approved the submitted version.

## Funding

This work received grant support (CORE project) from the Ministry for Science, Research and Arts of Baden-Württemberg, Germany and the European Commission (HORIZON2020 Project SUPPORT-E, 101015756) to HS, and from the German Red Cross Blood Transfusion Service Baden-Württemberg – Hessen to BJ.

## Conflict of Interest

The authors declare that the research was conducted in the absence of any commercial or financial relationships that could be construed as a potential conflict of interest.

## Publisher’s Note

All claims expressed in this article are solely those of the authors and do not necessarily represent those of their affiliated organizations, or those of the publisher, the editors and the reviewers. Any product that may be evaluated in this article, or claim that may be made by its manufacturer, is not guaranteed or endorsed by the publisher.
