# Supplementary material for: BNT162b2 Vaccination Elicits Strong Serological Immune Responses Against SARS-CoV-2 Including Variants of Concern in Elderly Convalescents
Source: Front Immunol. 2021 Sep 29;12:743422. doi: 10.3389/fimmu.2021.743422 (PMC8511403; doi:10.3389/fimmu.2021.743422)
Supplement: Supplementary file 1 [file DataSheet_1.pdf]

S1

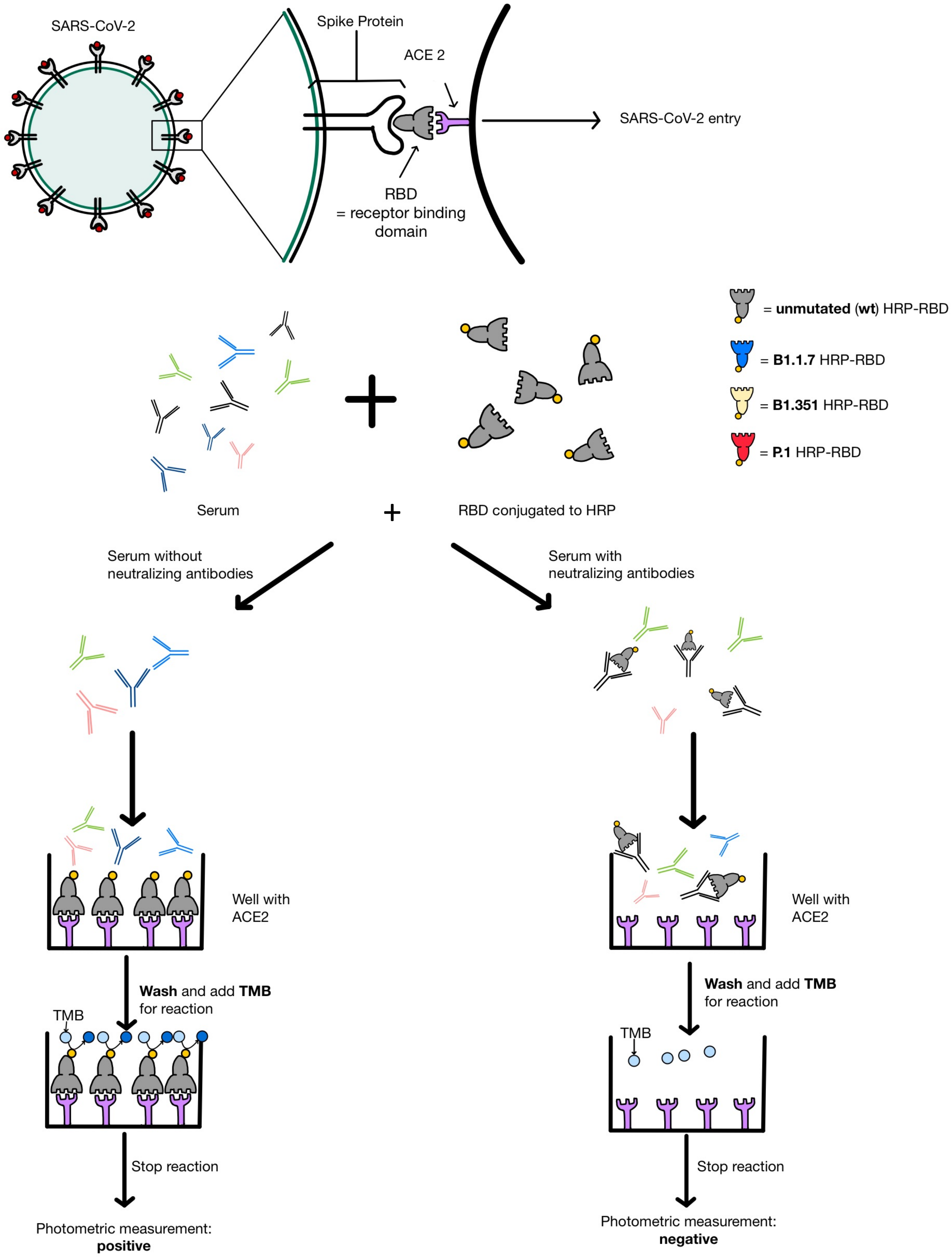

Supplementary Figure 1. Principle of the surroggate SARS-CoV-2 neutralization test

The ELISA system used to determine SARS-CoV-2 neutralization capacity mimics the cellular docking process of the virus through interaction between the SARS-CoV-2 receptor-binding domain (RBD) on the viral spike protein and the angiotensin-converting enzyme 2 (ACE2) on human cells. Unmutated or mutated variants of RBD are provided and used as horseradish peroxidase (HRP) conjugates (HRP-RBD). During a pre-incubation step, neutralizing antibodies in the serum of probands can bind to RBD-HRP conjugates. In a second step this mixture is added to microtiter plates coated with ACE2, allowing unblocked RBD-HRP conjugates to bind to ACE2. Finally, unbound HRP-RBD is washed away and remaining HRP catalyzes a TMB-based catalytic color reaction, which is photometrically measured. In the absence of neutralizing activity in the serum (left pathway) the chromogenic reaction is positive, whereas in the presence of neutralizing activity (right pathway) the reaction is suppressed. Abbreviations: ACE2: angiotensin-converting enzyme 2, HRP: horseradish peroxidase, RBD: receptor-binding domain, TMB: 3,3',5,5'-Tetramethylbenzidine.

S2A

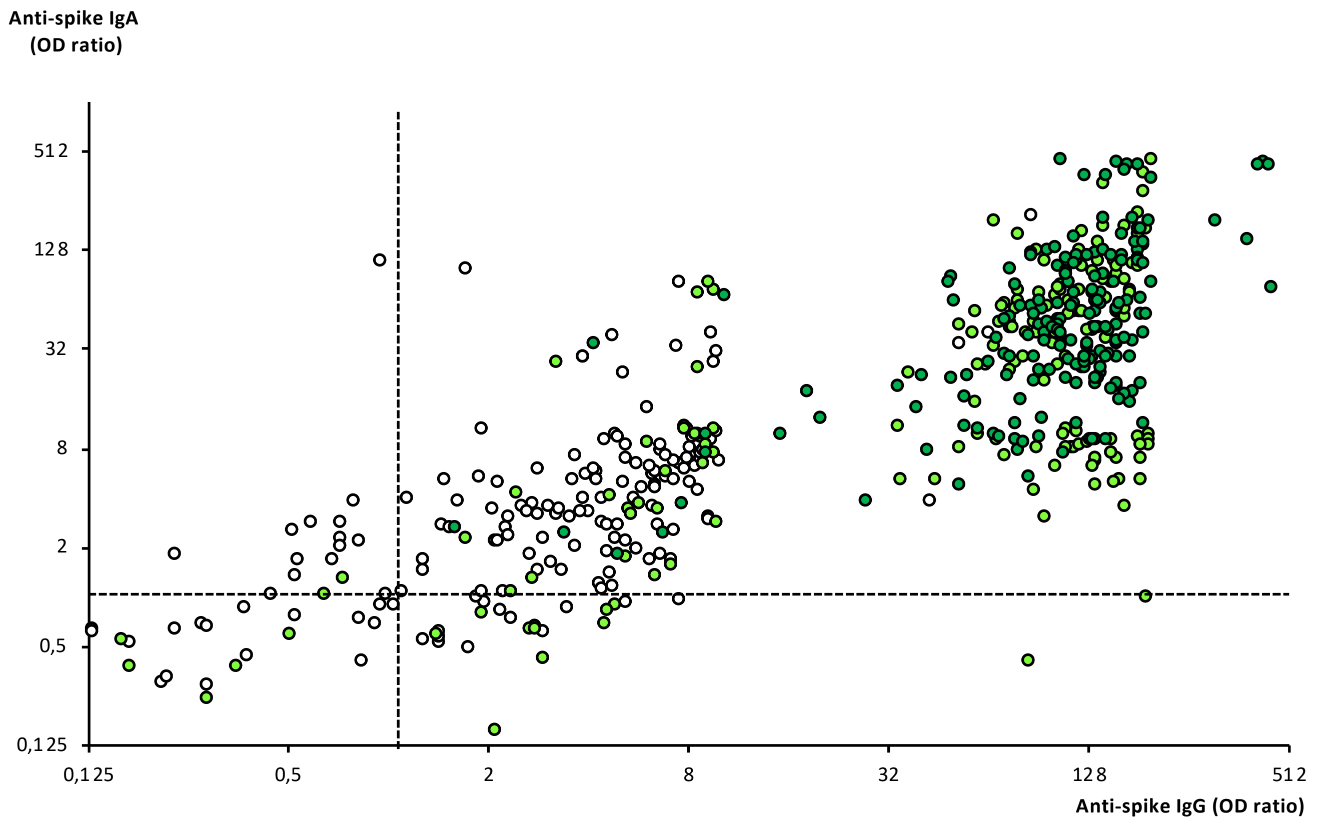

S2B

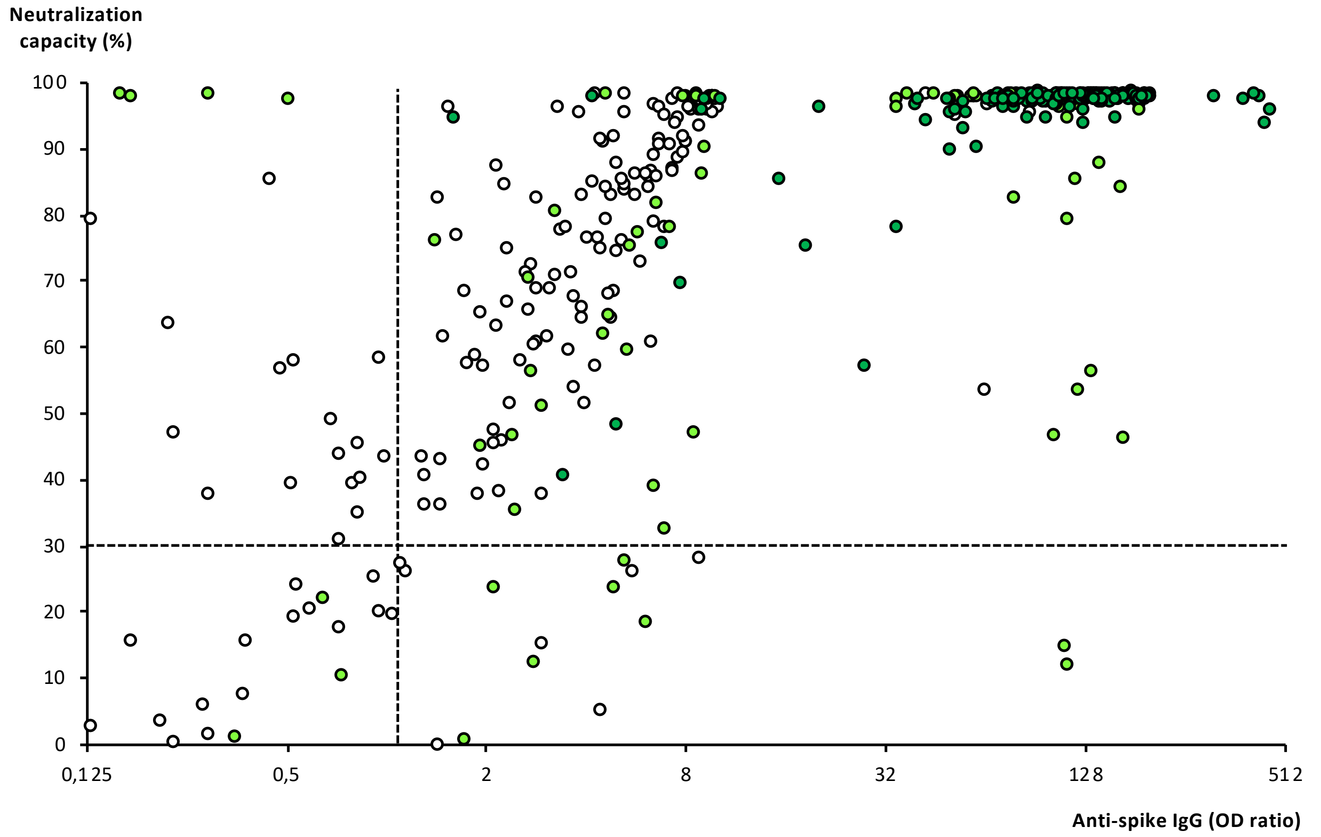

Supplementary Figure 2. Correlation between anti-SARS-CoV-2-spike antibody and ACE2-RBD neutralization titers. Serum samples were collected from a total of 190 individuals before vaccination (blank circles), follow-up samples from 181 subjects 3 weeks after first vaccination (bright green circles), and from 179 subjects 3 weeks after second vaccination (dark green circles) with BNT162b2. Samples were analyzed for anti-spike IgG and IgA titers as well as for ACE2-RBD neutralization capacities. Dot plots show (A) anti-spike IgG versus anti-spike IgA titers and (B) anti-spike IgG titers versus neutralization capacities. Cutoff titers for anti-spike IgG and IgA are set at an OD ratio of 1.1, cutoff for detectable neutralization capacity is set at 30% (dotted lines). Abbreviations: ACE2: angiotensin-converting enzyme 2, NCP: nucleocapsid, OD: optical density, RBD: receptor-binding domain.

S3

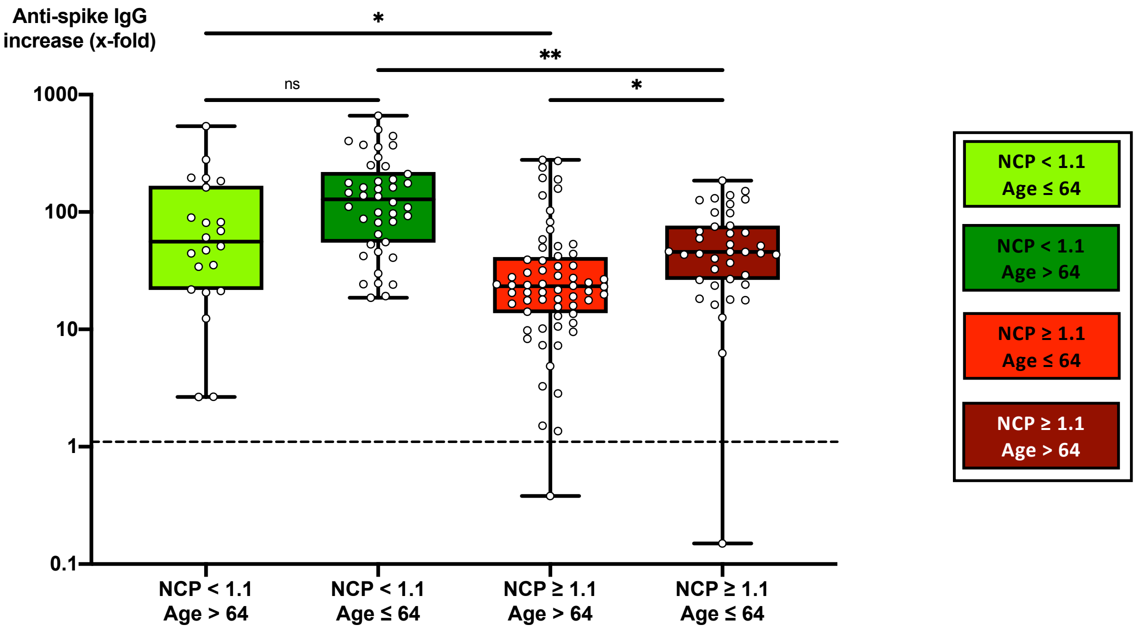

Supplementary Figure 3. Impact of age on the development of anti-spike IgG titers after vaccination.
Serum samples from 51 subjects with anti-NCP IgG OD ratios < 1.1 and an age < 64, from 22 subjects with anti-NCP IgG OD ratios < 1.1 and an age ≥ 64 , from 46 subjects with anti-NCP IgG OD ratios ≥ 1.1 and an age < 64 and from 71 subjects with anti-NCP IgG OD ratios ≥ 1.1 and an age ≥ 64 were collected before vaccination and 3 weeks after second vaccination with BNT162b2. Samples were analyzed for anti-spike IgG titers. Box blots show the x-fold increase of anti-spike IgG titers 3 weeks after vaccination compared to the titers before vaccination. Box central horizontal lines indicate medians, box borders represent IQR, whiskers indicate minima and maxima. Significance levels were * $p < 0.05$, ** $p < 0.005$. Abbreviations: IQR: interquartile ranges, ns: not significant.

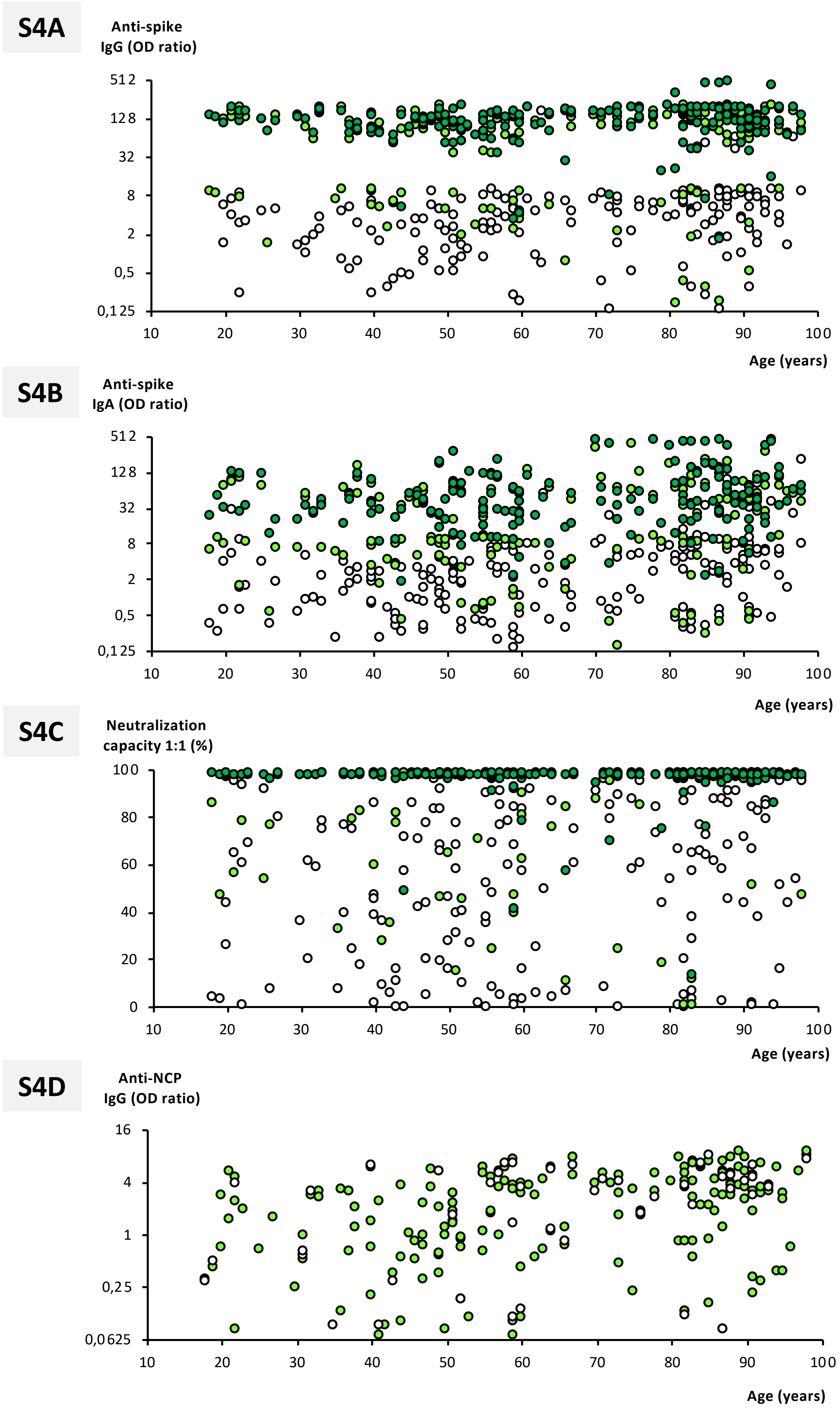

Supplementary Figure 4. Correlation between age and serological parameters.
Serum samples were collected from up to 190 individuals before vaccination (blank circles), follow-up samples from up to 181 subjects 3 weeks after first vaccination (bright green circles), and from up to 179 subjects 3 weeks after second vaccination (dark green circles) with BNT162b2. Samples were analyzed for anti-spike IgG (A), anti-spike IgA (B) and anti-NCP titers (D) as well as for ACE2-RBD neutralization capacities (C). Dot plots show correlations between age and the serological analytes described above. Abbreviations: NCP: nucleocapsid, OD: optical density.

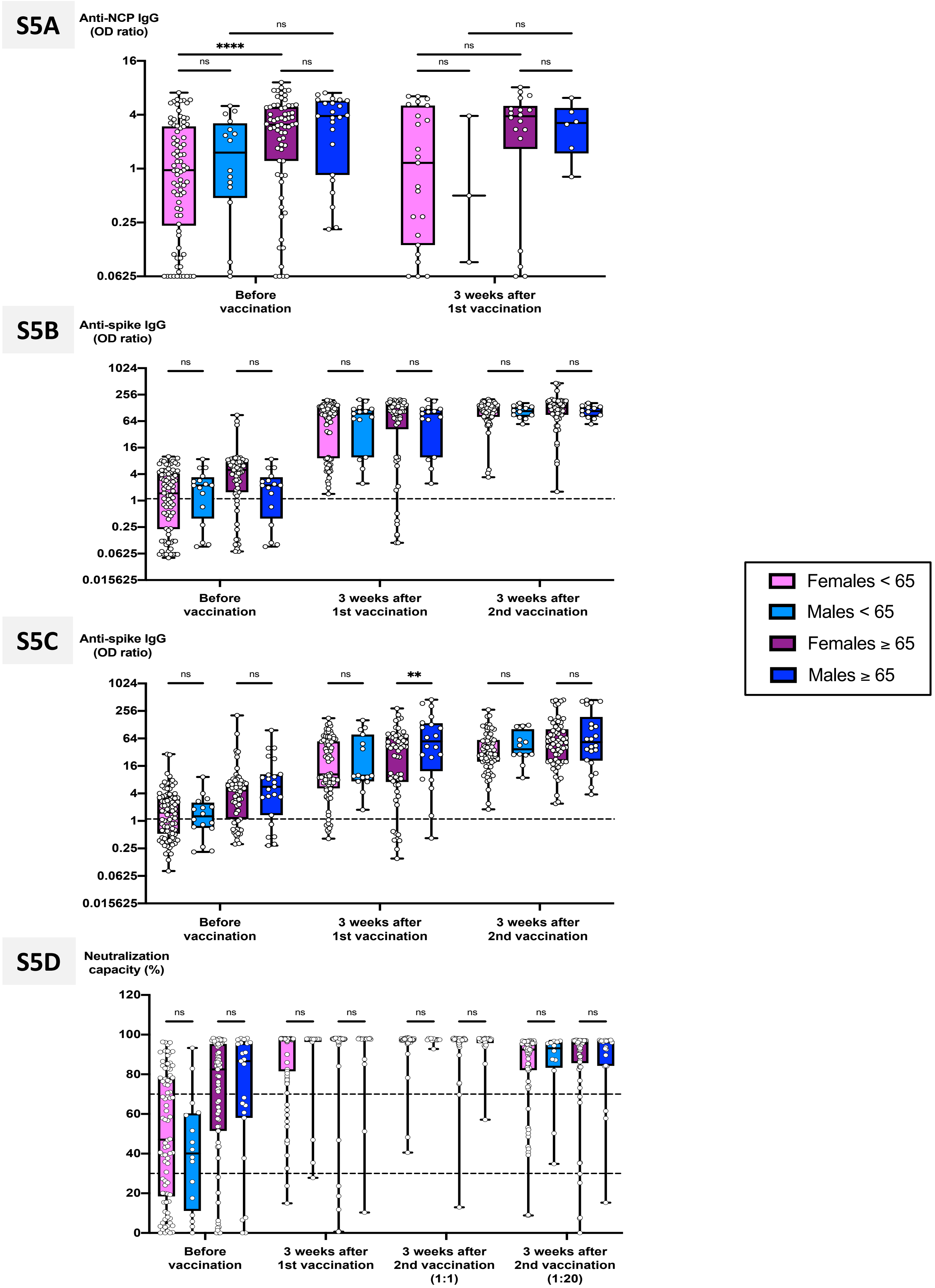

Supplementary Figure 5. Impact of gender on the development of humoral immune parameters after vaccination.
Serum samples from 82 females and 15 males with an age < 64, as well as from 67 females and 23 males with an age ≥ 64 were collected before vaccination, 3 weeks after first vaccination and 3 weeks after second vaccination with BNT162b2. Samples were analyzed for anti-spike IgG and IgA titers, anti-NCP IgG titers as well as for ACE2-RBD neutralization capacities. Box blots show (A) anti-NCP IgG titers, (B) anti-spike IgG titers, (C) anti-spike IgA titers and (D) median neutralization capacities at different time points as indicated. Dotted lines indicate cutoff values. To further differentiate high neutralization capacities 3 weeks after second vaccination, an additional analysis using an extra serum pre-dilution of 1:20 was performed (plot pair on the right side in panel D). Box central horizontal lines indicate medians, box borders represent IQR, whiskers indicate minima and maxima. Abbreviations: ACE2: angiotensin-converting enzyme 2, IQR: interquartile ranges, NCP: nucleocapsid, ns: not significant, OD: optical density, RBD: receptor-binding domain.
